# Characterization of Frictional Properties of Single-Layer Molybdenum-Disulfide Film Based on a Coupling of Tip Radius and Tip–Sample Distance by Molecular-Dynamics Simulations

**DOI:** 10.3390/nano8060387

**Published:** 2018-05-31

**Authors:** Haosheng Pang, Minglin Li, Chenghui Gao, Lianfeng Lai, Weirong Zhuo

**Affiliations:** 1 School of Mechanical Engineering and Automation, Fuzhou University, Fuzhou 350108, China; m150210010@fzu.edu.cn (H.P.); n160220004@fzu.edu.cn (W.Z.); 2 Fujian Key Laboratory of Medical Instrumentation and Pharmaceutical Technology, Fuzhou University, Fuzhou 350108, China; 3 Fujian Collaborative Innovation Center of High-End Manufacturing Equipment, Fuzhou University, Fuzhou 350108, China; 4 College of Information & Mechanical and Electrical Engineering, Ningde Normal University, Ningde 352100, China; lailianfeng82@163.com

**Keywords:** lateral force microscopy, MoS_2_, frictional properties, tip radius, vertical tip-sample distance

## Abstract

Lateral-force microscopy is a powerful tool to study the frictional properties of two-dimensional materials. However, few works distinctly reveal the correlation between the tip radius with the tip–sample distance and the frictional properties of the two-dimensional (2D) materials. We performed molecular-dynamics simulations to study the atomic-scale friction of a typical two-dimensional single-layer molybdenum disulfide (SLMoS_2_). The effects of tip radius and tip–sample distance on the frictional properties were analyzed and discussed. The frictional force–sliding-distance curves show typical stick–slip behaviors, and the periodicity can be used to characterize the lattice constants of SLMoS_2_. Sub-nanoscale stick-slip movements occur in one-lattice sliding periods along with only the armchair (AC) direction and only when the tip radius is smaller than 3 Å with 1.47 Å tip-sample distance. At the same tip–sample distance, a smaller tip can provide a more detailed characterization and higher-precision frictional properties of SLMoS_2_. A larger tip is capable of providing comparative frictional properties of SLMoS_2_ at a proper vertical tip–sample distance, compared with the small tip.

## 1. Introduction

Among the large family of scanning-probe microscopy techniques, lateral-force microscopy (LFM) is an effective tool to study the atomic-scale frictional behaviors of two-dimensional (2D) materials. LFM can be used to image insulating and conducting surfaces with atomic resolution, and record tip–sample force interactions with ultrahigh precision. This ability allows for atom-specific-force mapping with piconewton and picometer resolution [1,2]. Because experimental LFM measurements are affected by interatomic interactions between the probe tip and the sample surface [3], the tip features, including its stiffness [4], asymmetry [5,6], apex structure [7,8], and chemical identity [9,10], are important in lateral-force imaging of surfaces and the characterization of frictional behaviors at the atomic scale. In general, scanning-probe microscopy/LFM measurements are thought to be more accurate with a sharper tip [11,12]. Therefore, the effect of tip radius on the frictional properties has been usually ignored in previous studies. Besides, for the various component forces exerted by sample atoms on the tip such as Van der Waals (dispersion), Coulomb (electrostatic), dipole, and atomic forces, and the shapes of the force curves differing markedly [13], it is difficult to measure the tip–sample distance in the LFM experiments using only the LFM force curves. This approach has resulted in a lack of related studies on the effect of tip–sample distance on frictional properties. Hence, related parameters in the continuum mechanical model (for instance, Prandtl–Tomlinson model) of atomic-scale friction, such as the initial value of the tip–sample interaction, are usually set to empirical values [14,15,16]. To the best of our knowledge, no work exists that correlates the detailed tip radius with the tip–sample distance as a function of the frictional properties of the 2D materials.

Molecular dynamics (MD) simulation is a powerful method to reveal supplemental information in an endeavor to eliminate the limitations of LFM experiments. Various interaction potentials have been successfully developed to simulate materials such as ionic compounds [17], metals [18], and covalent materials [19]. The strength of MD simulation is that it can track the evolution of each atom’s configuration and provide a detailed mechanism of atomic-scale friction phenomena [20].

We conducted MD simulations of the atomic-scale friction of a typical 2D single-layer molybdenum disulfide (SLMoS_2_), which has been widely used in the applications of optoelectronics and electronics [21,22,23] due to its unique photoelectric and mechanical properties. The effects of tip radius and vertical tip–sample distance on the frictional properties were analyzed and discussed. Ours is the first study that indicates that the characterization of atomic-scale frictional properties depends on a coupling of tip radius and tip–sample distance, and the study presents novel insight into LFM nanomanipulation when studying the atomic-scale frictional behaviors of 2D materials.

## 2. Materials and Methods

SLMoS_2_ is composed of a hexagonal honeycomb lattice in which a layer of Mo atoms is sandwiched covalently between top and bottom layers of S atoms. The perpendicular distance between the two layers of S atoms is ~3.24 Å. A rectangular SLMoS_2_ film (230.31 Å × 259.61 Å in the x–y plane) was defined for MD simulations of sliding. The SLMoS_2_ film was fixed in all (x, y, and z) directions after system relaxation, and periodic boundary conditions were applied during the simulation.

**Figure 1:**
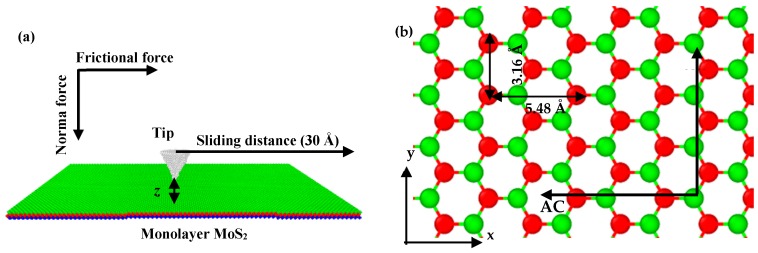
Schematic diagrams of the sliding on a SLMoS_2_ (**a**), lattice constants of SLMoS_2_ and sliding paths in various lattice orientations of SLMoS_2_ (**b**). Blue, red, and green spheres indicate S atoms (bottom layer), Mo atoms (middle layer), and S atoms (top layer) of SLMoS_2_, respectively, and z is the distance between the lowermost atoms of tip and the uppermost layer of S atoms.

The MD simulations were conducted by using a large-scale atomic/molecular massively parallel simulator (LAMMPs) (Albuquerque, NM, US) [24]. The interaction between carbon atoms in the diamond probe is described with the adaptive intermolecular reactive empirical bond-order (AIREBO) (Clemson, SC, US) potential which has been used to predict the mechanical properties of carbon structures in previous studies [25]. The atomic interactions between the SLMoS_2_ and the diamond probe are described by using the Lennard–Jones potential (Leigh, Lancashire, UK), which has been verified in our previous work [26]. The atomic interactions in SLMoS_2_ are described with reactive empirical bond-order (REBO) (Raleigh, NC, US) potentials, which have been demonstrated to be accurate when describing the mechanical properties of SLMoS_2_ [27]. Before the sliding process, the system was relaxed by energy minimization using the conjugated-gradient method and the time step was set to 1 fs. A schematic diagram of the sliding on SLMoS_2_ is shown in Figure 1a. Conical probes with various tip curvature radii of 1, 2, 3, 4, 5, 6, 7, 8, 9 and 10 Å (total atoms of 36, 291, 884, 2150, 4288, 7515, 12,021, 18,071, 25,846 and 34,302 respectively) were generated to study the effect of tip radius on the frictional properties of SLMoS_2_ film, and the sliding rate was set to 0.2 Å/ps. All the simulations were thermostated at 0.1 K.

**Figure 2:**
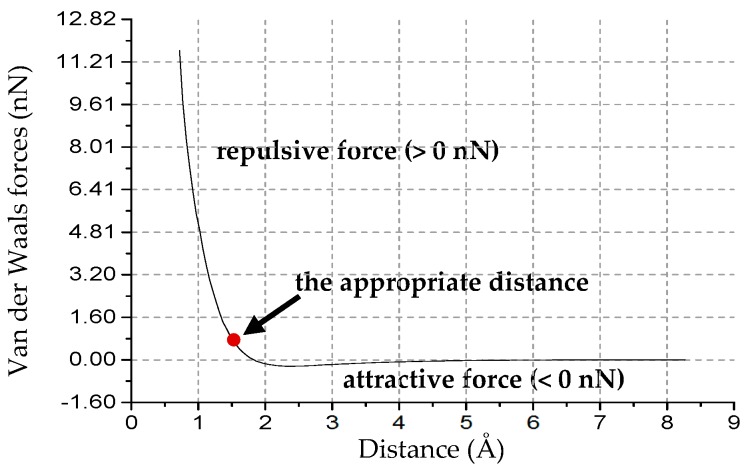
Van der Waals force–distance curve of the nanoindentation on SLMoS_2_. The repulsive force is given by larger than 0 nN, and the attractive force is smaller than 0 nN. The red point is the chosen reference point, which corresponds to the appropriate distance (1.47 Å) between the lowermost atoms of the tip and the uppermost layer of S atoms of SLMoS_2_.

The distance between the lowermost atoms of the tip and the uppermost layer of S atoms should be in an appropriate range for the following reasons: (1) If the distance is too large, the repulsive force of the tip-MoS_2_ interaction could be so weak that it may be significantly affected by thermal fluctuations; and (2) if the distance is too small, the uppermost atoms of MoS_2_ may collapse during the sliding process due to the large repulsive force. For instance, we performed an MD simulation of the nanoindentation on a SLMoS_2_, and the tip radius was set to 1 Å. We chose a reasonable tip–sample distance of 1.47 Å, which allows the tip to reach the near-contact region where the Pauli repulsion governs the tip-MoS_2_ interaction [28], and ensures that the tip is not very close to the uppermost MoS_2_ atoms. The van der Waals force–distance curve of the nanoindentation is shown in Figure 2. Besides, we also investigated the effect of tip–sample distance on the frictional properties with five vertical distances (1.27, 1.47, 1.67, 2.27, and 2.67 Å) and three different tip radii (1, 3, and 7 Å). The tip moves along the armchair (AC) and zigzag (ZZ) orientations. The lattice constants of the SLMoS_2_ and sliding paths are shown in Figure 1b.

We used the Open Visualization Tool (OVITO) (Darmstadt, Hesse-Darmstadt, Germany) to visualize and analyze atomistic simulation data.

## 3. Results and Discussion

**Figure 3:**
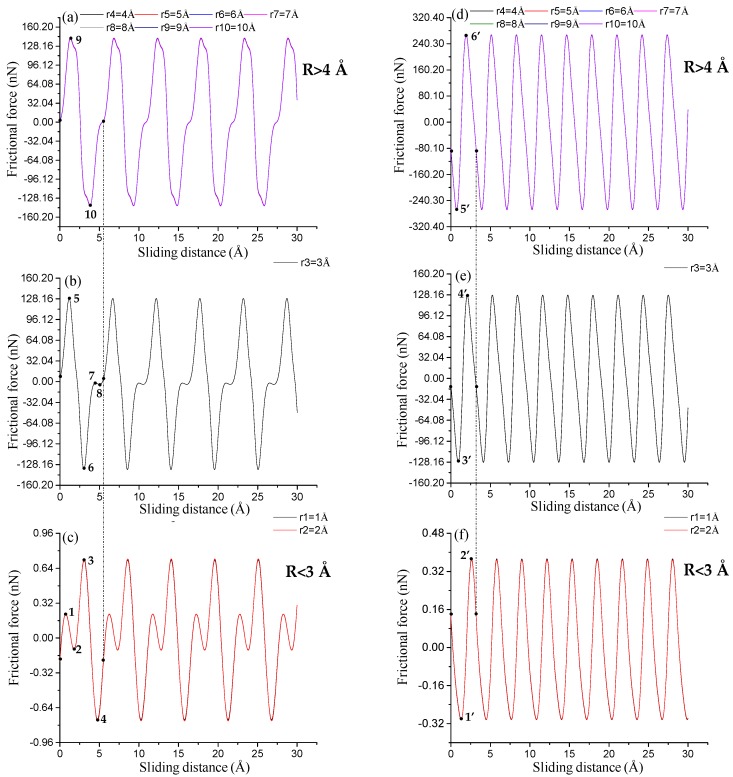
Frictional force–sliding distance curves for sliding paths along (**a**–**c**) AC orientation and (**d**–**f**) ZZ orientation of SLMoS_2_ for tips of 1 to 10 Å radii. Periodicities for each sliding path are almost constant for tips of all radii (1 to 10 Å radii), and are ~5.48 Å (**a**–**c**) for the AC orientation and 3.16 Å (**d**–**f**) for the ZZ orientation. The periodicities are the same as the lattice constants of SLMoS_2_. The r1, r2, r3, r4, r5, r6, r7, r8, r9 and r10 are defined as the tips with radii of 1, 2, 3, 4, 5, 6, 7, 8, 9 and 10 Å, respectively. The curves in Figure 3a,c,d,f look like a single curve, but they are the overlap of multiple curves, and the displayed curve is the dark colored curve (such as purple curves for Figure 3a,d, and red curves for Figure 3c,f).

All frictional force–sliding distance curves show typical stick–slip motions in atomic frictions (see Figure 3). The frictional forces exhibit sawtooth waves with the periodicity (i.e., adjacent spacings of peak frictional forces) of the MoS_2_ lattice. The periodicities of both sliding paths are capable of characterizing the lattice constants of SLMoS_2_ [14]. The periodicities for each sliding path are almost constant for all tips with radii of 1 to 10 Å, which is equal to ~5.48 Å (Figure 3a–c) for the AC orientation and 3.16 Å (Figure 3d–f) for the ZZ orientation. Moreover, sub-nanoscale stick–slip movements occur in one-lattice sliding periods along with only the AC direction and only when the tip radius is smaller than 3 Å (see the points of 1 to 4 in Figure 3c). Herein, the sub-nanoscale stick-slip behavior for SLMoS_2_ is obviously different to that for graphene [20], where sub-nanoscale stick and slip motions are found in the sliding along both the AC and ZZ directions.

**Figure 4:**
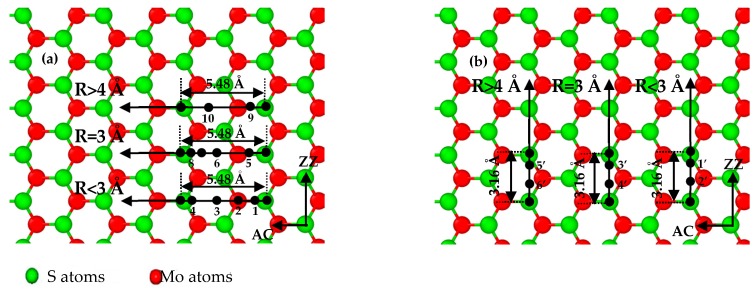
Sliding paths in various lattice orientations: black points are extreme points at frictional force–sliding distance curves in a single periodic length of (**a**) 5.48 Å for the AC orientation and (**b**) 3.16 Å for the ZZ orientation of SLMoS_2_ film.

**Figure 5:**
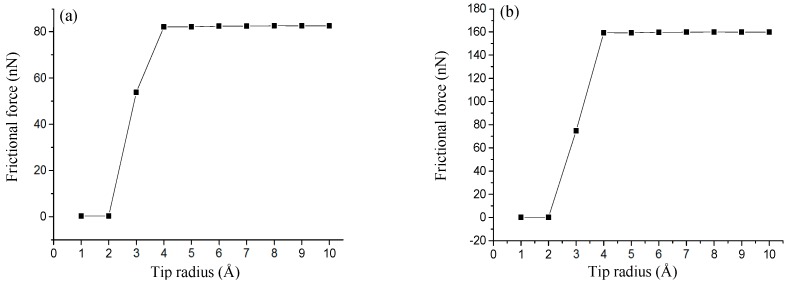
Variations of frictional forces as the tip slides on the surface of SLMoS_2_ for AC orientation (**a**) and ZZ orientation (**b**) with 10 different radii, 1, 2, 3, 4, 5, 6, 7, 8, 9, and 10 Å.

The sub-stick-slip behavior can be correlated with the lattice configuration. Figure 4 shows black extreme points on frictional force–sliding distance curves for AC and ZZ orientations in a single sliding periodicity. The difference in the number of extreme points in a single sliding periodicity results from the tip radius and lattice orientation, namely, two extreme points (i.e., points of 9 to 10) for an AC orientation and tips with radii of 4 to 10 Å (Figure 3a), four extreme points (i.e., points of 1 to 4, and points of 5 to 8) for an AC orientation and tips with radii of 1 to 2 Å (Figure 3b), and 3 Å (Figure 3c), and two extreme points (i.e., points of 1’ to 2’ in Figure 3f, points of 3’ to 4’ in Figure 3e, and points of 5’ to 6’ in Figure 3d) for a ZZ orientation and tips of all radius, where the separation distance between the adjacent extreme points expose the information of lattice structure. The frictional forces increase with an increase in tip radius, as shown in Figure 5, where the small amplitude of the frictional force is suggested to expose the detailed information of frictional properties. The frictional force amplitudes for tips with radii of 1 to 2 Å are much smaller than those for tips with radii of 4 to 10 Å. 

**Figure 6:**
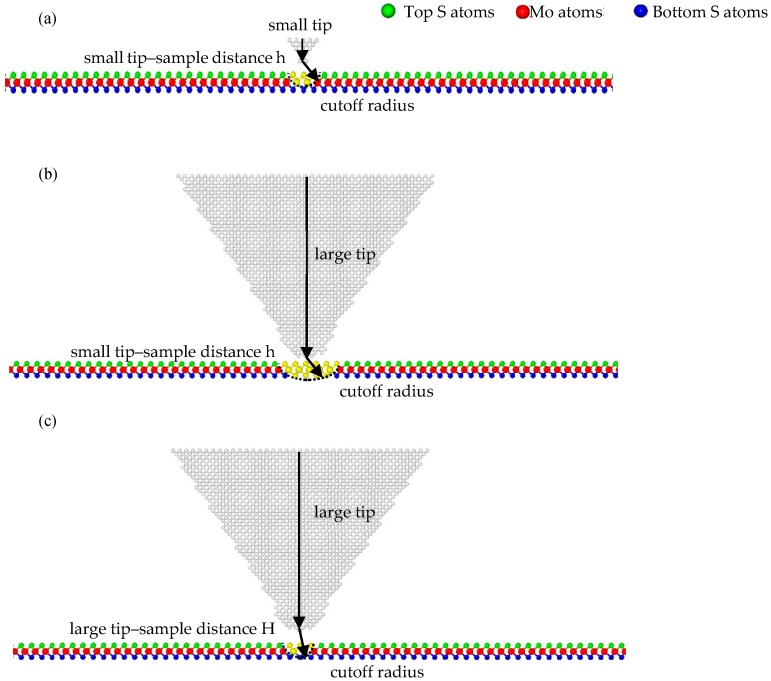
Schematic diagrams for tips of various radius during sliding: (**a**) small tip at small tip–sample distance h; (**b**) large tip at small tip–sample distance h; (**c**) large tip at large tip–sample distance H. Yellow regions represent the range of influence of the Van der Waals force for tips with various radii.

There is one viewpoint [15] that the sub-nanoscale stick-slip motions stem from the 2D scanning path and depend on the lattice orientation of the substrate, which can be explained by the 2D Prandtl–Tomlinson model. They found that moving trajectories of the tip in the AC and ZZ orientations are the main reason for the differences in details (the number of extreme points and the wave shape) of the frictional force–sliding distance curves of LFM tips on SLMoS_2_. In addition, some unexpected sub-nanoscale stick-slip motions usually occur in the curves during the LFM experiments because the tip stiffness is so small (0.02 N/m), and the movement of the tip is affected by the load [4]. However, our simulation results show that 2D scanning paths are not necessarily required for the sub-nanoscale stick-slip motion, under the condition that the smaller tip radii are employed when the tip-substrate distance is specified. We attribute its underlying mechanism to the number of interactive atoms, as illustrated in Figure 6. The larger tips contain more carbon atoms than the smaller tips, when the cutoff for carbon atoms in the Lennard–Jones potential is constant. Therefore, the influence of the van der Waals force of the larger tip is larger than that of the small tip. In other words, the small tip is more susceptible to the interactive forces of surrounding atoms of SLMoS_2_ during the sliding process; however, the influence of interactive forces for the larger tip is not so remarkable, as shown in Figure 6a,b. Logically possible, large tips (3 to 10 Å) can provide the comparative detailed and higher-precision frictional properties (e.g., sub-nanoscale stick slip motions) of SLMoS_2_ when reducing the interactive atom number by increasing the tip–sample distance, as shown in Figure 6c.

**Figure 7:**
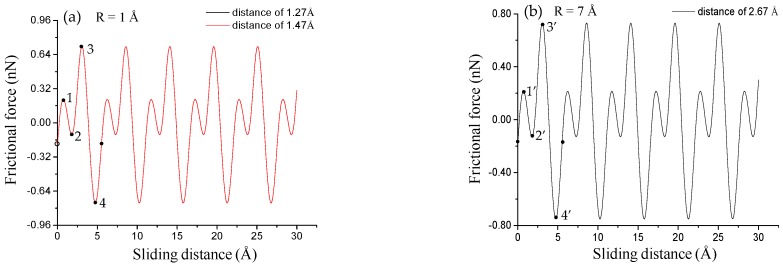
Frictional force–sliding distance curves for various vertical distances (1.27, 1.47 and 2.67 Å) between the tip and SLMoS_2_ film for sliding paths along the AC orientation. The tip radii of 1 Å, and 7 Å are for (**a)** and (**b)**, respectively. All the frictional force–sliding distance curves for various vertical distances (1.27, 1.47, 1.67, 2.27, and 2.67 Å) between the tip and SLMoS_2_ film for sliding paths along the AC orientation and ZZ orientation are shown in Figure S1 (Supplementary Materials).

Herein, we conducted MD simulations of LFM tip sliding on SLMoS_2_ with various tip radius (1, 3, and 7 Å) and tip–sample distances (1.27, 1.47, 1.67, 2.27, and 2.67 Å). The stick–slip behaviors of all frictional force–sliding distance curves are shown in Figure 7 and Figure S1). The curves in Figure 7a are the overlap of multiple curves, and the displayed curves are the red curve, which are the same as that in Figure 3. Increasing the tip-substrate distance results in the decrease of the frictional force for all tip radii. For the tips with radius larger than 2 Å, there are some appropriate tip-sample distances that will lead to the detailed frictional properties (e.g., sub-nanoscale stick slip motion) comparable with that of less than 2 Å tip radii. Take the tip with 7 Å (or 3 Å) for example shown in Figure 7b (or Figure S1f, Supplementary Materials), the frictional force–sliding distance curve with a tip-sample distance of 2.67 Å (2.27 Å or 1.67 Å) is as good as that of 1 Å tip with the distances of 1.27 Å and 1.47 Å shown in Figure 7a. These similarities may result because the Van der Waals force of the larger tip at a proper large tip–sample distance is almost equal to that of the small tip, as shown in Figure 6b,c. Results in Figure 7 and Figure S1 (Supplementary Materials) indicate that as long as the vertical tip–sample distance is controlled, the larger tip (e.g., 7 Å radius) is able to provide the detailed frictional properties of SLMoS_2_ comparable to that of the small tip (1 Å radius), which has not been reported in any other studies thus far. 

Furthermore, we would suggest that the adiabatic potential [15,29] in the 2D Prandtl–Tomlinson model can be modified to reveal the effect of a combination of tip radius and tip-sample distance on the frictional properties of SLMoS_2_-like 2D materials, as shown in Equation (1). The adiabatic potential presents the interaction between the tip and the surface of SLMoS_2_, where $\alpha_{x}$ and $\alpha_{y}$ are the lattice constants of the S layer in a MoS_2_ film. *V*_0_$\left(Z_{tip-sample}\right)$ depends on the scanning height *Z_tip-sample_* above the MoS_2_ surface, and it decreases rapidly when *Z_tip-sample_* exceeds an atomic distance [29,30]. $A\left(R_{tip},Z_{tip-sample}\right)$ is the coupling term considering the interweaving of the tip radius ($R_{tip}$) and the tip-sample distance ($Z_{tip-sample}$). These coefficients could be characterized quantitatively by many more simulations and experiments in future investigations. The results can provide guidance and enlightenment for sub-nanoscale stick–slip motions.

(1) $$V\left(x_{t},y_{t}\right)=A\left(R_{tip},Z_{tip-sample}\right)\cdot V_{0}\left(Z_{tip-sample}\right)\cdot \cos\left(\frac{2\pi }{a_{x}}x_{t}\right)\cdot \cos\left(\frac{2\pi }{a_{y}}y_{t}\right)$$

**Figure 8:**
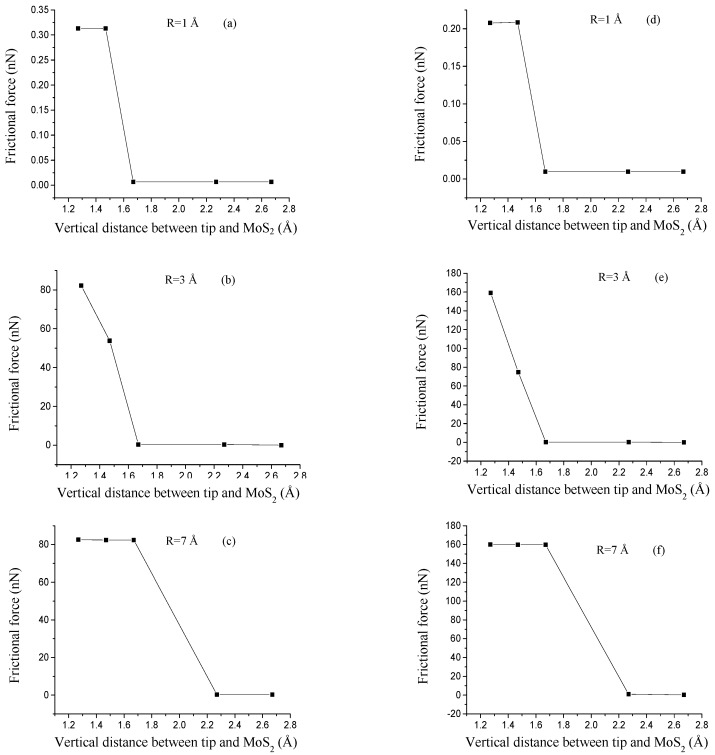
Variations in frictional forces for three different tips (radii of 1, 3, and 7 Å) sliding along the AC (**a**–**c**) and ZZ (**d**–**f**) orientation with five vertical distances between tips and MoS_2_, z = 1.27 Å, z = 1.47 Å, z = 1.67 Å, z = 2.27 Å, and z = 2.67 Å, respectively.

Besides the tip radius of 7 Å with the tip-sample distance of 2.27 Å and 2.67 Å (Figure S1k,l, Supplementary Materials), the amplitudes of frictional force for all the tips increase with the decrease of the tip-sample distance. We calculate the average frictional forces, as shown in Figure 8. The average frictional forces increase with a decrease in tip MoS_2_ distance z, which means that a smaller vertical distance z results in a stronger repulsive interaction between the tip and MoS_2_, and it is more difficult for the tip to slide on the MoS_2_ surface. The results agree with those in the research literature [30,31]. We also found that the variation trends of frictional forces for the AC and ZZ orientation are different. It can be due to the different interactive atoms of moving trajectories. As shown in Figure 4, sliding paths are S-Mo-S atom for AC orientation and S-S atom for ZZ orientation, respectively. Besides, sub-nanoscale stick-slip movements are found in the sliding process along with only the AC orientation, which indicates the chirality effect of frictional properties of SLMoS_2_, as shown in Figure 3 and Figure S1 (Supplementary Materials).

## 4. Conclusions

MD simulations were conducted on the atomic-scale friction of SLMoS_2_ film. All frictional force–sliding distance curves show typical stick–slip behaviors, and their periodicity can characterize the lattice constants of the MoS_2_. Sub-nanoscale stick-slip movements occur in one-lattice sliding periods along with only the AC direction and only when the tip radius is smaller than 3 Å with 1.47 Å tip-sample distance. A smaller tip can provide more detailed and higher-precision frictional properties of SLMoS_2_ at the same tip–sample distance. The frictional force curves of various vertical tip–sample distances with tips of different radius show that as long as the tip–sample distance is set reasonably, a larger tip also describes the frictional properties of the SLMoS_2_, and achieves the precision range of the small tip. This study presents novel insight into the LFM manipulation that correlates various tip radii with different vertical tip–sample distances and the dependence of frictional properties of SLMoS_2_ film. The results from this study provide reference for LFM experiments and indicate that a sharp tip is unnecessary at a reasonable tip–sample distance.

## Supplementary Materials

The following are available online at http://www.mdpi.com/2079-4991/8/6/387/s1, Figure S1: Frictional force–sliding distance curves for various vertical distances (1.27, 1.47, 1.67, 2.27, and 2.67 Å) between the tip and SLMoS2 film for sliding paths along the AC orientation (a–b, e–g, and k–m) and ZZ orientation (c–d, h–j, and n–p). The tip radii of 1 Å, 3 Å, and 7 Å are for a–d, e–j, and k–p, respectively. The curves in Figure S1 (a), (b), (c), (d), (f), (i), (m) and (p) are the overlap of multiple curves, and the displayed curves are the dark colored curve (such as purple curves for Figure S1 (a), (c), (m) and (p), and red curves for Figure S1 (b), (d), (f) and (i), which are the same as that in Figure 3.
