# Supplementary material for: Characterization of Frictional Properties of Single-Layer Molybdenum-Disulfide Film Based on a Coupling of Tip Radius and Tip–Sample Distance by Molecular-Dynamics Simulations
Source: Nanomaterials (Basel). 2018 May 31;8(6):387. doi: 10.3390/nano8060387 (PMC6027478; doi:10.3390/nano8060387)
Supplement: Supplementary file 1 [file nanomaterials-08-00387-s001.pdf]

SUPPORTING MATERIALS

1. **Figure S1.** Frictional force–sliding distance curves for various vertical distances (1.27 Å, 1.47 Å, 1.67 Å, 2.27 Å, and 2.67 Å) between the tip and SLMoS2 film for sliding paths along the AC orientation (a–b, e–g, and k–m) and ZZ orientation (c–d, h–j, and n–p). The tip radii of 1 Å, 3 Å, and 7 Å are for a–d, e–j, and k–p, respectively. The curves in Figure S1 (a), (b), (c), (d), (f), (i), (m) and (p) are the overlap of multiple curves， and the displayed curves are the dark colored curve (such as purple curves for Figure S1 (a), (c), (m) and (p), and red curves for Figure S1 (b), (d), (f) and (i)), which are the same as that in Figure 3.

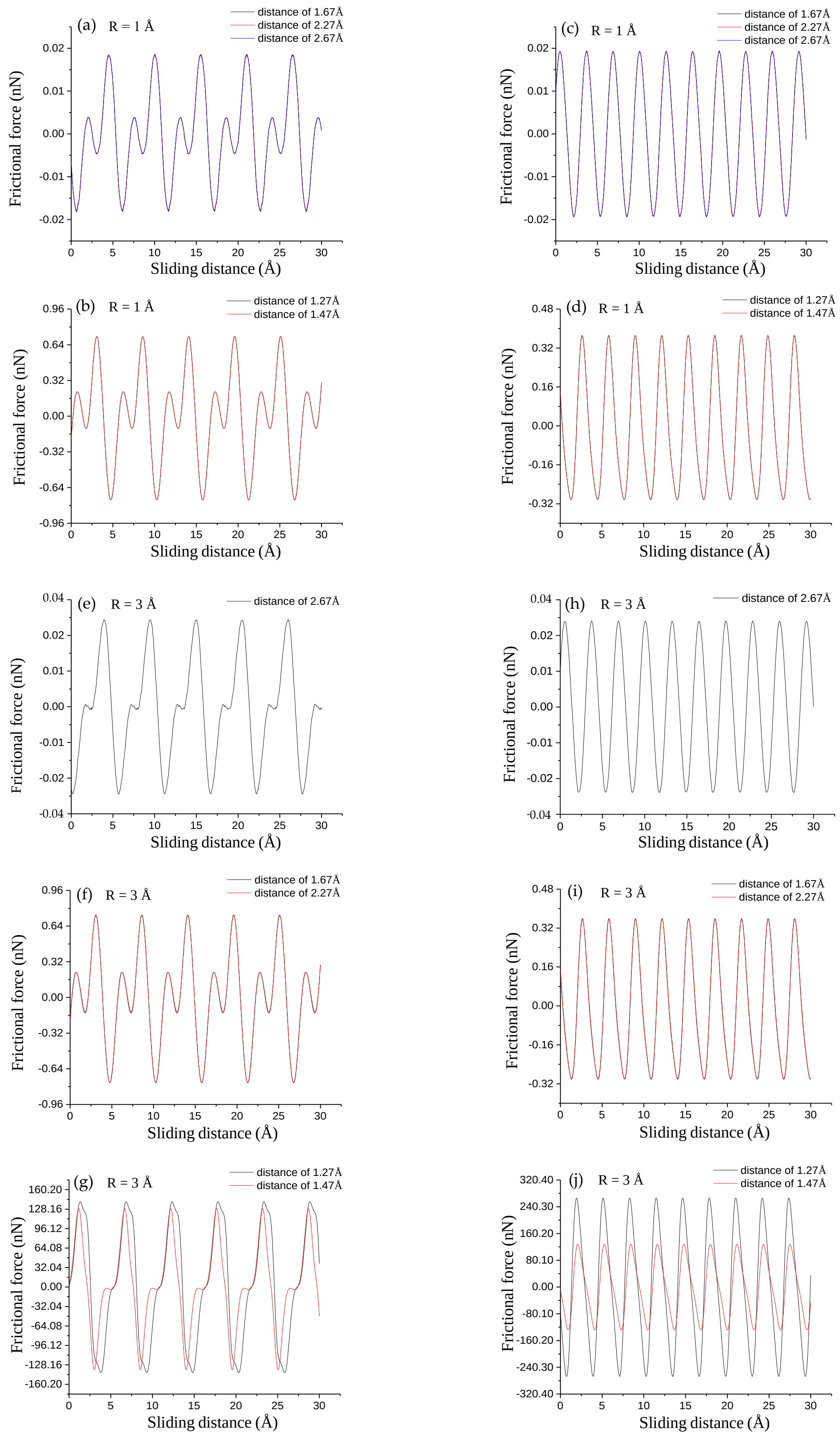

(a) R = 1 Å
distance of 1.67Å
distance of 2.27Å
distance of 2.67Å
Frictional force (nN)
Sliding distance (Å)
(c) R = 1 Å
distance of 1.67Å
distance of 2.27Å
distance of 2.67Å
Frictional force (nN)
Sliding distance (Å)
(b) R = 1 Å
distance of 1.27Å
distance of 1.47Å
Frictional force (nN)
Sliding distance (Å)
(d) R = 1 Å
distance of 1.27Å
distance of 1.47Å
Frictional force (nN)
Sliding distance (Å)
(e) R = 3 Å
distance of 2.67Å
Frictional force (nN)
Sliding distance (Å)
(h) R = 3 Å
distance of 2.67Å
Frictional force (nN)
Sliding distance (Å)
(f) R = 3 Å
distance of 1.67Å
distance of 2.27Å
Frictional force (nN)
Sliding distance (Å)
(i) R = 3 Å
distance of 1.67Å
distance of 2.27Å
Frictional force (nN)
Sliding distance (Å)
(g) R = 3 Å
distance of 1.27Å
distance of 1.47Å
Frictional force (nN)
Sliding distance (Å)
(j) R = 3 Å
distance of 1.27Å
distance of 1.47Å
Frictional force (nN)
Sliding distance (Å)

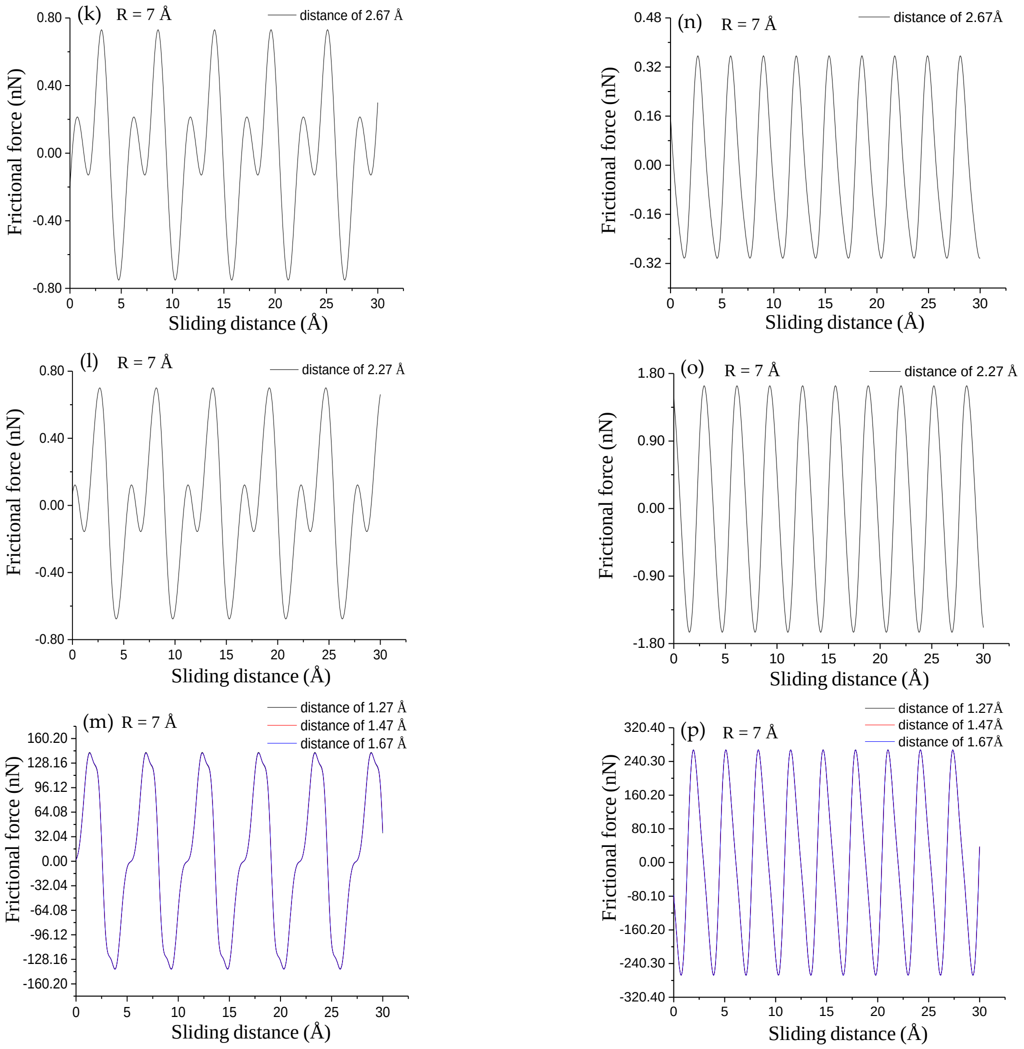
(k) R = 7 Å
distance of 2.67 Å
0.80
0.40
0.00
-0.40
-0.80
Frictional force (nN)
0 5 10 15 20 25 30
Sliding distance (Å)
(n) R = 7 Å
distance of 2.67Å
0.48
0.32
0.16
0.00
-0.16
-0.32
Frictional force (nN)
0 5 10 15 20 25 30
Sliding distance (Å)
(l) R = 7 Å
distance of 2.27 Å
0.80
0.40
0.00
-0.40
-0.80
Frictional force (nN)
0 5 10 15 20 25 30
Sliding distance (Å)
(o) R = 7 Å
distance of 2.27 Å
1.80
0.90
0.00
-0.90
-1.80
Frictional force (nN)
0 5 10 15 20 25 30
Sliding distance (Å)
(m) R = 7 Å
distance of 1.27 Å
distance of 1.47 Å
distance of 1.67 Å
160.20
128.16
96.12
64.08
32.04
0.00
-32.04
-64.08
-96.12
-128.16
-160.20
Frictional force (nN)
0 5 10 15 20 25 30
Sliding distance (Å)
(p) R = 7 Å
distance of 1.27Å
distance of 1.47Å
distance of 1.67Å
320.40
240.30
160.20
80.10
0.00
-80.10
-160.20
-240.30
-320.40
Frictional force (nN)
0 5 10 15 20 25 30
Sliding distance (Å)
